# Changing role of the amygdala in affective and cognitive traits between early and late adulthood

**DOI:** 10.3389/fpsyt.2023.1033543

**Published:** 2023-02-07

**Authors:** Gaelle E. Doucet, Jordanna A. Kruse, Noah Hamlin, Jacob J. Oleson, Stuart F. White

**Affiliations:** ^1^Institute for Human Neuroscience, Boys Town National Research Hospital, Omaha, NE, United States; ^2^Department of Pharmacology and Neuroscience, Creighton University School of Medicine, Omaha, NE, United States; ^3^Department of Biostatistics, University of Iowa, Iowa City, IA, United States

**Keywords:** aging, amygdala, anxiety, fMRI, verbal fluency, affective Stroop

## Abstract

**Introduction:**

Healthy aging is typically associated with cognitive decline and lower negative affect. Previous studies have reported a significant and opposite role of the amygdala in relation to cognitive and affective processing in early adulthood. However, it remains unclear how aging impacts such relationships.

**Methods:**

Seventy-seven healthy participants including 40 young (mean age = 26.1 years) and 37 older (mean age = 61.8 years) adults completed a functional MRI Affective Stroop (AS) paradigm, a cognitive battery, and the state-trait anxiety inventory. The AS fMRI paradigm included “task trials,” where participants saw a positively, negatively or neutrally valenced distractor image, followed by a numerical display, followed by another distractor image. We extracted signal in both amygdalas during the AS Task and compared it across all conditions and age group. We further conducted moderation analyses to investigate the impact of aging on the relationship between amygdala activation and anxiety or cognitive variables, respectively.

**Results:**

At the behavioral level, older participants showed lower trait anxiety than the younger adults (*p* = 0.002). While overall slower during the AS task, older adults achieved comparable accuracy during the AS task, relative to the younger adults. At the brain level, we revealed a significant interaction between age group and trial types in amygdala activation (*F* = 4.9, *p* = 0.03), with the older group showing stronger activation during the most complex trials compared to the passive view trials. We further found that age significantly modulated the relationship between anxiety and the left amygdala activation during negative stimuli, where the younger adults showed a positive association while the older adults showed a negative association. Age also significantly modulated the relationship between verbal fluency and left amygdala activation during incongruent versus view trials, with the younger adults showing a negative association and the older adults showing a positive association.

**Discussion:**

The current study suggests that the role of the amygdala on both emotional processing and cognitive traits changes between early and late adulthood.

## 1. Introduction

Healthy aging is typically associated with some level of cognitive decline (1, 2) and improved emotional well-being, particularly lower negative affect (3–6). With regards to changes in emotional well-being, behavioral studies have extensively reported that aging is inversely related to specific experiences of negative affect, such that older adults typically report lower anger and anxiety (7). Such age-related emotional changes have been linked to the socioemotional selectivity theory (SST) which suggests that motivational priorities shift across the lifespan as a function of future time horizons (8). However, while the neural mechanisms behind this theory remain relatively unclear, it is strongly suggested to be linked with alterations of the amygdala. In this context, two major concepts have been raised (6, 9): one involved an age-related decline in brain regions that monitor negative stimuli, such as the amygdala (10). The other model, more supported by experimental and neuroimaging findings, suggest that older adults use different regulation strategies than younger adults, largely relying of the prefrontal cortex and its interaction with the amygdala (5, 6, 9). The main evidence for this latter theory is based on various magnetic resonance imaging (MRI) studies that have reported no significant difference in amygdala activation in response to fearful (11) or surprised (12) vs. neutral faces, as well as very little change in the structural integrity of the amygdala (6), between older and younger adults. Mather (6) further suggested that the “amygdala does not stop responding to emotional stimuli in later life, but instead, shifts which valence it is most responsive to” (p. 218).

Regardless of the theory, the amygdala remains a key region of interest to understand changes in affective processing. However, in addition to its well-known role in emotional processing and particularly acute threat (13, 14), the amygdala has also been linked to cognitive and attentional processes based on both human and non-human studies (15, 16). Using functional MRI (fMRI), a recent study conducted on the Human Connectome Project-Young Adult dataset reported that higher amygdala activation during a working memory task was associated with lower cognitive performance in young adults (17). Further, the amygdala has been shown to respond differently as a function of cognitive demand (6, 17, 18), as well as anxiety (19, 20). Overall, such associations have been largely investigated in early adulthood and it remains unknown how the amygdala activity during an affective processing task may be influenced by anxiety, cognitive decline and cognitive loads in older adults, relative to younger adults.

In this context, the current study aimed to investigate the impact of healthy aging on the amygdala function during emotional and attentional processing, and how it interacts with anxiety and cognitive abilities. To do so, 81 healthy participants, including 41 young adults (aged 19–35) and 40 older adults (aged 50–81), were recruited and completed an affective Stroop (AS) paradigm while undergoing functional MRI. Each participant also completed the NIH cognitive battery and the State-Trait Anxiety Inventory (21). We further quantified amygdala activation in response to either emotional stimuli (positive, negative or neutral) or varying cognitive load (incongruent, congruent, view) and its association with anxiety and cognitive traits, respectively, in younger and older adults. We hypothesized that: (1) amygdala response to emotional stimuli will vary by cognitive loads, (2) the amygdala would respond less strongly to negative stimuli in the older group while there will be no difference in activation in the amygdala between the two age groups while viewing positive stimuli, (3) age will modulate the relationship between anxiety and amygdala activation during emotional processing, and (4) age will also significantly impact the association between higher-order cognitive abilities and amygdala activation during attentional processing.

## 2. Materials and methods

### 2.1. Participants

We recruited 81 healthy adults, which were further categorized in a younger adult group [*n* = 41; mean age (SD) = 26.2 (3.5) years, age range: 20.8–33.6 years; 17 males] and an older adult group [*n* = 40; mean age (SD) = 62.8 (7.0) years, age range: 51.5–81.9 years; 18 males]. Exclusion criteria in the study included any chronic medical illness affecting central nervous system function, any neurological or psychiatric disorder, acute intercurrent illness, pregnancy, history of head trauma, current substance use disorder, and presence of any ferrous metal implant which may interfere with the MRI data acquisition. Four subjects were further excluded from analyses because of poor performance during the AS fMRI scan (overall accuracy < 80%); which resulted in a total of 40 younger adults [age = 26.1 (3.5) years, 17 males] and 37 older adults [age = 61.8 (6.1) years, 16 males]. Groups did not significantly differ in sex (*p* = 0.97), handedness (*p* = 0.66), or education level (*p* = 0.64) (Table 1). However, the older adults had a lower Mini-Mental State Examination (MMSE) score [younger: mea*n* = 29.4 (0.7), range: (26–30); older: mea*n* = 28.8 (1.1), range: (25–30), *p* = 0.02]. The study was approved by the Institutional Review Board for Research with Human Subjects at Boys Town National Research Hospital. Each participant provided written informed consent.

**Table 1:** Demographic information for each age group.

	**Younger participants** **(*n =* 40)**	**Older participants** **(*n =* 37)**	**Statistics**
Age, mean (std) (years)	26.07 (3.53)	61.84 (6.13)	*T =*−31.6, *p < * 0.001
Age range (years)	20.86–33.58	51.49–74.73	
Sex, *n* females (%)	23 (57.5%)	21 (56.8%)	X^2^ = 0.2, *p =* 0.99
MMSE, mean (std)	29.4 (0.74)	28.84 (1.11)	*T =* 2.8, *p =* 0.016
Education, mean (std) (years)	16.38 (1.05)	16.54 (1.88)	*T =* −0.8, *p =* 0.558
State-STAI Range	31.03 (7.68) 20–56	29.43 (6.79) 20–50	*T =* 1.0, *p =* 0.346
Trait-STAI Range	37.28 (8.04) 20–56	31.14 (8.04) 20–52	*T =* 3.3, *p =* 0.002
Total STAI Range	68.3 (14.95) 40–112	60.57 (13.64) 40–88	*T =* 2.4, *p =* 0.020
Verbal fluency^*^ Range	48.20 (11.20) 19–71	43.03 (9.75) 26–67	*T =* 2.2, *p =* 0.034
Fluid intelligence (*t*-score) Range	54.9 (9.14) 36–73	53.44 (8.93) 35–72	*T =* 0.7, *p =* 0.486
Crystallized intelligence (*t*-score) Range	54.63 (8.91) 39–80	51.42 (6.41) 36–68	*T =* 1.8, *p =* 0.078

MMSE, Mini-Mental State Examination; STAI, State-Trait Anxiety Inventory. ^*^Based on the total number of words said starting by the letters F, A or S, in one min each. All continuous variables are reported as mean (standard-deviation).

### 2.2. Anxiety and cognitive assessment

To assess anxiety level, each participant completed the State–Trait Anxiety Inventory (STAI) (21). Current anxiety levels were measured with the A-State and long-term anxiety with the A-Trait scale. A high score measured with the STAI stands for a high anxiety level. Fluid and crystallized intelligence measures were assessed using the cognitive battery from the NIH Toolbox (https://www.healthmeasures.net/explore-measurement-systems/nih-toolbox). Fluid intelligence represents the composite performance score on tasks of memory, executive function and processing speed; crystallized intelligence represents the composite performance score on tasks on picture vocabulary and oral reading recognition. The t-scores (i.e., corrected for age, sex, ethnicity/race, and education) of each variable were used for further analyses. Lastly, verbal fluency was assessed as the sum of total number of words a participant verbalizes starting with a F, A and S in 1 min each.

### 2.3. MRI data acquisition

Participants were scanned on a 3T Siemens Prisma scanner using a 32-channel head coil. Structural images were acquired using a T1-weighted, 3D magnetization-prepared rapid gradient-echo (MPRAGE) sequence with the following parameters: Repetition Time (TR) = 2,400 ms, Echo Time (TE) = 2.05 ms, Field of View (FOV): 256 × 256 mm, 1 mm isotropic resolution, Inversion Time (TI) = 1,000 ms, 8° flip angle, bandwidth = 240 Hz/Pixel, echo spacing = 7.0 ms, in-plane acceleration GRAPPA (GeneRalized Autocalibrating Partial Parallel Acquisition) factor 2, total acquisition time ~6 min. Participants also completed two runs of an affective Stroop task, using a multi-band T2^*^ sequence with the following acquisition parameters: TR = 480 ms, TE = 29.2 ms, voxel size = 3 × 3 × 3 mm^3^, 44° flip angle, echo spacing = 0.51 ms, bandwidth = 2,772 Hz/Pixel, number of axial slices = 56, multi-band acceleration factor = 8, duratio*n* = 4 min 9 s. For each run, 503 volumes were collected.

### 2.4. Affective Stroop fMRI task

**Figure 1:**
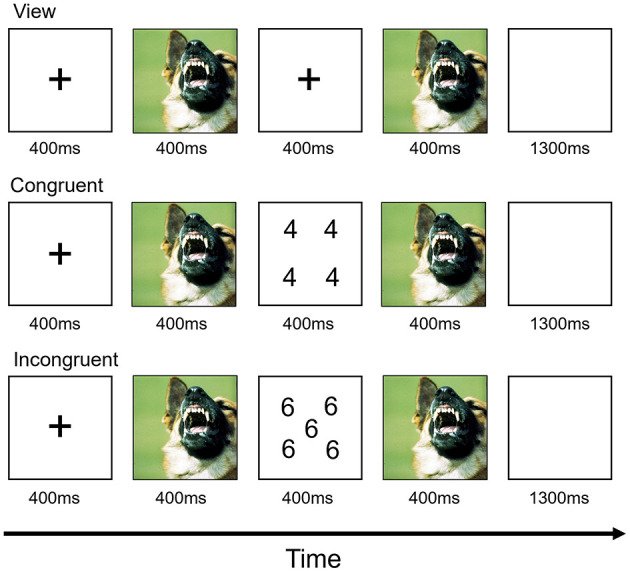
Design of the affective Stroop fMRI task.

The AS task used here was an adapted version of a paradigm described previously (22, 23). Each trial began with a fixation cross centrally presented for 400 ms (Figure 1). This was followed by a 400 ms image presentation. In view trials, participants were then presented with a blank screen for 400 ms. During task trials, a numerical display was presented for 400 ms. For both view and task trials, there was then a second 400 ms period during which the first image was presented again. This was followed by a 1,300-ms blank screen. The subjects had to determine the quantity of digits in the numerical display. That is, how many of the numbers were displayed, not the actual value of the numbers. For congruent trials, the quantity of numbers displayed was the same as the number value (e.g., five 5's and six 6's). For incongruent trials, the quantity of numbers displayed did not equal the number values (e.g., four 5's and five 3's). Participants could respond at any time from the presentation of the numerical display until the end of the blank screen. View trials required no response.

The individual numerical stimuli consisted of three, four, five, or six 3 s, 4 s, 5 s, or 6 s randomly presented within a 9-point grid (Figure 1). Within each run, the emotional stimuli consisted of 32 positive, 32 negative, and 32 neutral pictures selected from the International Affective Picture System (24). The normative mean valence and arousal values on a 9-point scale were, respectively, 3.35 ± 0.77 and 5.97 ± 1.07 for negative pictures, 7.43 ± 0.52 and 4.99 ± 1.10 for positive pictures, and 4.87 ± 0.28 and 2.66 ± 0.54 for neutral pictures. There were nine trial types: view, congruent, and incongruent trials involving negative, positive, and neutral emotional stimuli. Subjects completed two runs, each consisting of 16 trials of each of the nine trial classes and 48 fixation-point trials to generate a baseline. Each image was presented once in a congruent trial, once in an incongruent trial and once in a view trial. Each image appeared only in one run. Trials were randomized within each run for each participant and counterbalanced between participants. Accuracy and reaction times (RTs) collected during the task were extracted for each participant. Behavioral responses were analyzed using repeated-measured ANOVAs with emotions (positive, negative and neutral) and conditions (congruent and incongruent) being two within-subject factors and age group entered as the between-network factor.

### 2.5. FMRI preprocessing

The fMRI data were preprocessed using Statistical Parametric Mapping (SPM12) and the DPABI Toolbox (25). For both runs, preprocessing procedures included motion correction to the first volume with rigid-body alignment; co-registration between the functional scans and the anatomical T1-weighted scan; spatial normalization of the functional images into Montreal Neurological Institute (MNI) stereotaxic standard space; and spatial smoothing within the functional mask with a 6-mm at full-width at half-maximum Gaussian kernel. Because maximum volume-to-volume head motion was under 2 mm or degrees for all participants, no participants were excluded for this reason.

### 2.6. FMRI task activation

General linear model analyses were implemented using SPM12. For each run, the preprocessed single-participant images were analyzed in a similar fashion, using a linear convolution model. Nine regressors were generated using an event-related design, and included in the model: (i) negative congruent, (ii) negative incongruent, (iii) negative view, (iv) neutral congruent, (v) neutral incongruent, (vi) neutral view, (vii) positive congruent, (viii) positive incongruent and (ix) positive view. There were also regressors of no-interest for incorrect trials (1) and head motion (6). All regressors were created by convolving the train of stimulus events with a gamma variate hemodynamic response function to account for the slow hemodynamic response.

At the second level, we specifically extracted mean parameter estimate from the right and left amygdalas, using the AAL atlas (26) and the Marsbar toolbox (27) for each condition. We conducted a repeated-measures ANOVA to identify the main effects of age group (younger vs. older; between-subjects), trial types (congruent, incongruent and view; within-subject), emotions (positive, negative, neutral; within-subject), hemispheres (right and left; within-subject) and all pairwise interactions, followed-up by *post-hoc* analyses when appropriate. Statistically significant results were reported after applying a Family Discovery Rate (FDR) correction. The ANOVAs were conducted in SPSS v25.

Based on previous studies (17, 22) and our hypotheses, we further investigated the associations between amygdala activation and (1) anxiety and (2) cognitive measures (three measures: verbal fluency, fluid and crystallized intelligence), and the impact of aging on these, by conducting moderation analyses. The contrasts used for the amygdala activations were selected based on the results from the previous analysis (repeated-measures ANOVA, see result section). Significant results are reported at a FDR-corrected level. The moderation analyses were conducted using the *lm* function in R version 4.0.3.

### 2.7. Supplementary analyses

While not the main topic of this study, we also conducted whole brain analyses and report results for full report of completeness. At the whole-brain level, we conducted one-sample *t*-tests on the beta images resulting from the first-level model estimation, to identify the specific networks responding to: (1) emotional stimuli using the contrast: positive+negative view > neutral view; and (2) executive function using the contrast: congruent+incongruent trials > view. Significant networks were identified using a whole brain threshold of *p* < 0.05 [family wise error (FWE) corrected] and a minimum number of voxels at the cluster level of >10. Age, sex and mean head motion (i.e., mean framewise displacement) were added as covariates. For each contrast, two sample *t*-tests were also conducted to identify spatial differences between the two age groups. Sex and mean head motion were added as covariates. Significant differences were reported at a whole brain threshold of *p* < 0.001, and a cluster-level *p* < 0.05 after applying a FWE correction.

## 3. Results

### 3.1. Behavioral results

**Figure 2:**
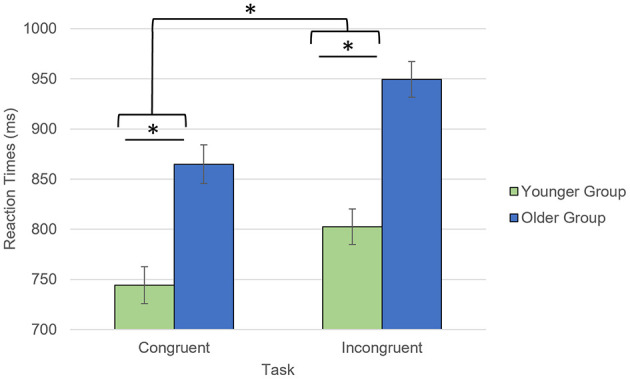
Task by group interaction for the reaction times collected during the AS task. *p < 0.05. Reaction times for accurate responses were considered.

Older participants reported lower anxiety than the younger participants, which was driven by trait anxiety (trait: *t* = 3.29, *p* = 0.002, state: *t* = 0.97, *p* = 0.34; Table 1). During the AS task, both groups showed high accuracy for both congruent (Younger: 97.4%; Older: 96.7%, *p* = 0.09) and incongruent trials (Younger: 94.3%; Older: 94.2%, *p* = 0.30). Regarding the RTs, there was a significant interaction between age group and trial type (*F* = 6.7, *p* = 0.012) with the older participants being slower for the incongruent than congruent trials, compared to the younger participants (Figure 2). While the main effects of age group (older being slower than the younger participants; *F* = 33.1, *p* < 0.001) and trial type (slower RTs for incongruent than for congruent trials; *F* = 218.5, *p* < 0.001) were also significant, there was no significant main effect of emotional valence on the RTs (*F* = 0.41, *p* = 0.53) or emotional valence by group interaction (*F* = 0.70, *p* = 0.41).

### 3.2. ROI analyses

**Figure 3:**
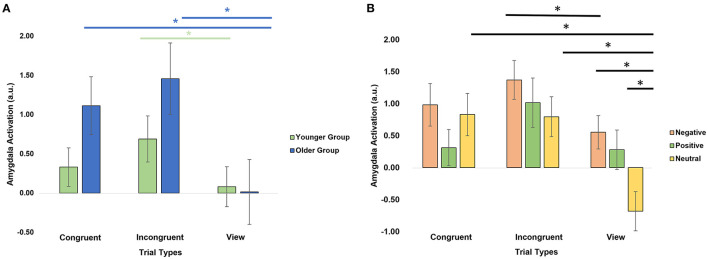
Amygdala activation by trial types. **(A)** Significant age group × trial type interaction. **(B)** Significant emotion × trial type interaction. *Significant *post-hoc* analyses at *p* < 0.05 after applying a FDR correction.

We further investigated the level of activation in the amygdala across trial types by conducting a repeated-measures ANOVA with within-subject factors hemisphere (right, left), emotional valence (negative, positive, neutral), trial type (congruent, incongruent, view) and age group as a between-subject factor (Table 2). There was a significant trial type × age group interaction (*F* = 4.9, *p* = 0.03, Figure 3A). *Post-hoc* analyses revealed that the older group activated the amygdalas more during the incongruent (*t* = 4.06, *p*_FDR_ = 0.002) and congruent (*t* = 3.35, *p*_FDR_ = 0.006) trials than during the view trials. The younger group showed more limited differences with stronger activation in the incongruent trials only (*t* = 2.58, *p*_FDR_ = 0.028), compared to the view trials.

**Table 2:** Repeated-measures ANOVA on the amygdala activation.

**Source**	**Mean square**	***F***	**Sig**.
Hemisphere	0.144	0.012	0.914
Hemisphere × age group	1.037	0.085	0.771
**Emotion**	**97.817**	**16.646**	**<0.001**
Emotion × age group	1.356	0.231	0.632
**Trial types**	**104.888**	**12.416**	**0.001**
**Trial types × age group**	**41.470**	**4.909**	**0.030**
Hemisphere × emotion	5.537	3.211	0.077
Hemisphere × emotion × age group	0.006	0.004	0.952
Hemisphere × trial types	1.180	0.658	0.420
Hemisphere × trial types × age group	0.680	0.379	0.540
**Emotion × trial types**	**45.031**	**5.496**	**0.022**
Emotion × trial types × age group	0.032	0.004	0.950
Hemisphere × emotion × trial types	0.055	0.055	0.816
Hemisphere × emotion × trial types × age group	1.679	1.684	0.198

Significant variables at *p* < 0.05 are shown in bold.

There was also a significant emotion × trial type interaction (*F* = 5.50, *p* = 0.02, Figure 3B). *Post-hoc* paired t-tests revealed that the amygdalas activated more strongly during the Negative View (*t* = 4.39, *p*_FDR_ = 0.0003), Positive View (*t* = 3.13, *p*_FDR_ = 0.01), Negative Incongruent (*t* = 2.75, *p*_FDR_ = 0.029), Neutral Congruent (*t* = 4.45, *p*_FDR_ = 0.0003), and Neutral Incongruent (*t* = 4.08, *p*_FDR_ = 0.0007) trials than the Neutral View trials, and during Negative Incongruent than during Negative View trials (*t* = 2.75, *p*_FDR_ = 0.029). Lastly, significant main effects of emotions (*F* = 16.6, *p* = 1.11E-04; Negative > Positive > Neutral) and trial types (*F* = 12.4, *p* = 0.001; Incongruent > Congruent > View) were also revealed (Table 2).

**Figure 4:**
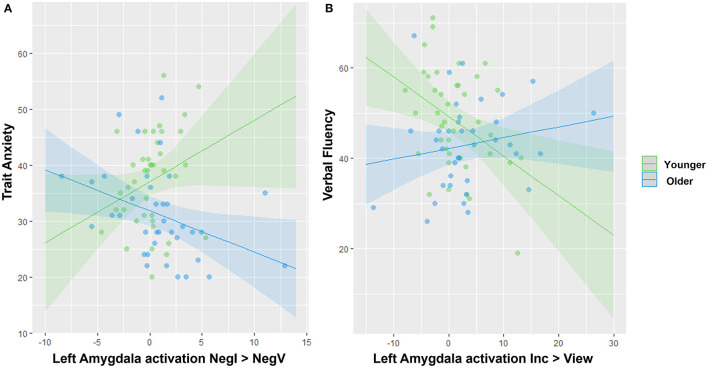
Significant interactions between age group and amygdala activation to predict anxiety or cognitive traits. **(A)** Opposite associations between amygdala activation and trait anxiety in younger vs. older adults. **(B)** Opposite associations between amygdala activation and verbal fluency in younger vs. older adults. Note that removing the two blue outliers in **(A)** resulted in a stronger age effect.

Lastly, we conducted moderation analyses to investigate the impact of age on the association between amygdala activation and either anxiety or cognitive variables. In regard to anxiety, we specifically focused on trait anxiety because it was significantly different between the two groups (Table 1), and for the contrasts, we focused on the three showing the strongest differences in amygdala activation involving a negative or positive valence (Figure 3): Negative View—Neutral View, Positive View—Neutral View, and Negative Incongruent—Negative View. We also investigated right and left amygdala separately. We found a significant interaction between age group and the left amygdala activation for the Negative Incongruent vs. Negative View contrast (*t* =−2.67, *p*_FDR_ = 0.038; Figure 4A). In detail, in the younger participants, higher anxiety was positively associated with amygdala activation (β = 1.09, 95% CI = −0.11, 2.29) while this relationship was negative in the older participants (β = −0.734, 95% CI = −1.38, −0.09). No significant interactions with the other contrasts were revealed, even at an uncorrected threshold.

For the moderation analyses involving cognitive variables, we tested the amygdala activation during the significant contrasts reflecting different levels of cognitive loads with the Incongruent vs. View and Congruent vs. View contrasts only (Figure 3). We revealed a significant interaction between age group and the left amygdala activation during the Incongruent vs. View trials to predict verbal fluency (*t* = 2.81, *p*_FDR_ = 0.037; Figure 4B). In detail, younger adults showed a negative association (β = −0.875, 95% CI = −1.52, −0.23) while the older adults showed a positive one (β = 0.237, CI = −0.22, 0.69). We did not reveal any other significant modulation by age group between amygdala activation and cognitive variables, even at an uncorrected level.

These results (significant interactions with age) remained significant even after adding MMSE score in each respective model.

### 3.3. Supplementary analyses

Whole brain activation for the positive+negative view > neutral view and congruent+incongruent trials > view contrasts, respectively, across all participants are reported in Supplementary Figure S1 and Supplementary Table S1. When viewing pictures with emotional valence compared to neutral valence, participants showed typical bilateral activation in amygdala, and along the fusiform gyri and the lateral occipital cortex (Supplementary Figure S1A). There was no significant difference between the two age groups. The second contrast (Supplementary Figure S1B) largely revealed the attentional network with bilateral activation along the intraparietal sulcus, frontal eye field, supplementary motor area, cerebellum, subcortical regions and insulas. The older group showed stronger activation in lateral parietal cortices, partially overlapping with the sensorimotor network and cerebellar regions, compared to the younger group (Supplementary Table S1).

## 4. Discussion

The current study investigated the impact of healthy aging on (1) the amygdala responses during an affective Stroop fMRI task and (2) the association of amygdala activation with anxiety and cognitive traits. In line with the positivity effect (28), we described that our older participants reported less anxiety than the younger participants. At the brain level, amygdala activation was modulated by the level of cognitive load, with this being more noticeable in the older than in the younger participants. We also described a modulation of the response of amygdala by emotional valence with a stronger response during negative stimuli, than positive, than neutral stimuli, especially during the passive viewing condition. While we expected an attenuation of amygdala response to negative stimuli in the older group (6, 9), we could only detect a small trend for the passive view trials, compared to the younger group (Supplementary Figure S2). Lastly, our moderation analyses revealed a significant impact of age on the association between amygdala activation and cognitive and emotional traits. Notably, higher anxiety in younger adults was associated with stronger activation of the left amygdala during negative stimuli while the older adults showed the inverse relationship. In contrast, higher verbal fluency in younger adults was related to lower activation in the left amygdala during the incongruent vs. view trials, while this was the inverse in the older group.

One of the main findings of the current study is the interaction between task conditions and the age group, with stronger amygdala activation in conditions requiring larger cognitive loads and working memory capacity in older than in younger. Based on previous studies done on adolescents and young adults, we did not expect to show such pattern as amygdala has typically been described as more active during passive conditions (6, 18, 22). A possible explanation is that the task—originally designed to be conducted in children and adolescents (29)—was not complex enough for healthy adults, as evidenced by high accuracy rates in every condition and both groups. This may indicate that the level of cognitive loads required to complete this task remained relatively lower compared to other cognitive tasks tested in previous neuroimaging studies (9). Consistently with this idea, the network revealed during the congruent + incongruent > view contrast (Supplementary Figure S2) revealed regions typically involved in attentional processing rather than during cognitive control [i.e., larger activation in the parietal lobe and low-to-no activation in the dorsolateral prefrontal cortex which would be typically the case in the executive control network (30)]. The current pattern of findings suggests that increased effort/attention was needed to process all the stimuli during the congruent and incongruent trials, but that “representational competition” (31) did not occur using the current paradigm. It is possible that a faster or more complex presentation of the stimuli was needed for adults in order to generate an interference effect.

Regardless, the finding that the older group activated more strongly the amygdala to emotional stimuli during mildly challenging conditions, than the younger group, further suggests that the amygdala function may not be impaired in older adults and therefore also does not support the aging-decline model (10). However, it should be noted that our findings are also not totally consistent with the cognitive-control model as well, as we did not find significant differences in amygdala activation in response to negative stimuli between the two age groups. Nonetheless, given the strong interaction revealed between the trial conditions and the age groups, and the fact that the two active conditions involved some level of attentional processing, we believe the current findings are more in line with the hypothesis from the cognitive-control model describing an increased interaction between the two brain systems (limbic vs. cognitive control) with aging. While our analyses were solely focused on the amygdala, which is a central region of the limbic system, it will be important that future analyses include other regions of the system, such as anterior cingulate and insula as well as regions from the cognitive control system through connectivity analyses.

Furthermore, it is interesting to note that we did not reveal a significant interaction between age and emotional valence in the amygdala despite evidence of that the amygdala becomes relatively more responsive to positive than negative stimuli in aging (5, 6). However, Moriguchi et al. (32) have also reported a lack of valence by age interaction. Therefore, more studies are definitely needed to conduct a more thorough investigation using different cognitive tasks, different cognitive loads and emotional valences on amygdala activation with larger sample sizes to clarify the origins of these discrepancies.

In line with our hypotheses, we found a moderation of the relationship between the amygdala activation and anxiety or cognitive traits by age. First, higher anxiety was associated with higher amygdala activation in younger but not older adults. Such finding in young adults has been consistently described across functional studies in both adolescents (33–35) and younger adults (35, 36) with anxiety disorders. Anxiety disorders have been typically associated with dysregulation, particularly through an over-activation, of the amygdala in response to the presentation of emotional stimuli, compared to individuals with low anxiety symptoms [see meta-analysis by Etkin and Wager (20)]. This positive association has been suggested to reflect an exaggerated engagement of the fear circuit during emotional processing (20). To our knowledge, though, little is known about the relationship between anxiety traits and amygdala activity in older populations (healthy or diagnosed with late-life anxiety disorders). Our older group was overall less anxious than the young adults while showing similar level of amygdala activation. A possible interpretation is that amygdala responsiveness to emotional stimuli may not be as responsive to anxiety in late adulthood as it is in early life, which could be caused by an increasing interaction with the prefrontal cortex (6, 9). We further believe that it could reflect an aging-related functional reorganization of the neural correlates responding to anxiety. Future studies with larger sample sizes and longitudinal data should be conducted to investigate this hypothesis.

While the amygdala's role has been widely investigated in the context of emotional processing, several studies have more recently reported its role in working memory and stimuli detection (15, 17, 37). Particularly, it has been proposed that the successful suppression of amygdala activity happens as resources are routed to prefrontal regions for cognitive tasks via top-down inhibition, as shown by a negative relationship between amygdala activation during working memory tasks and cognitive performances (17, 38, 39). Consistently, another study has reported that individuals with an amygdalar lesion show higher working memory performance (40). Our findings on young adults are consistent with these studies, as we also described that lower amygdala activation during the incongruent vs. view trials is associated with higher verbal fluency. In contrast, older adults showed a positive association. Given that our older sample reported slightly lower verbal fluency than our younger sample (although the mean values were within one standard deviation from each other, Table 1), this indicates that the older adults with fluency scores closer to those reported by the younger adults, showed stronger amygdala activation during the most complex cognitive condition. Importantly, these results remain significant even after accounting for difference in MMSE across groups. This may be interpreted as maintained cognitive abilities in older age may be related to a reduction in top-down inhibition from the frontal lobe to the amygdala during complex cognitive processing. In fact, previous studies in older populations have suggested that decline in executive function may be more related to structural and functional changes in the ventromedial areas of the prefrontal cortex (41), which is a region with substantial functional and structural connections with the amygdala [e.g., (42)].

While this study has many strengths, we should acknowledge some limitations. First, our analyses were based on cross-sectional samples and not longitudinal data. As discussed by the revised scaffolding theory of aging and cognition model (43), investigating the rate of within-subject change in cognitive and affective processing is essential to understand and identify brain integrity preservation vs. compensatory mechanisms which may support preserved cognition in older adults. Future studies should also investigate the impact of structured behavioral interventions on the association between amygdala activation and cognitive and affective traits in late adulthood, using different cognitive and affective tasks. Second, our sample size was modest and it will be important to test the reproducibility of our findings in a larger independent sample and across the whole adulthood range. Third, it is possible that the findings described in the younger group were driven by the participants with higher anxiety scores. While this issue could be resolved by using a sample matched on anxiety scores, this could have the unintended effect of distorting the aging sample. As described by the “positivity effect” (28), older adults often show reduced anxiety relative to younger adults, which has been described as an *effect* of aging. By removing this normal aspect of aging from the sample, we would risk studying a sample that did not reflect normal aging, particularly in the light of the fact that no participants met criteria for any psychiatric disorder. Fully understanding this issue will require longitudinal studies. Relatedly, older participants that are volunteering to a research study are likely to be healthier and more energetic than non-volunteering older adults. This potential confound will also be best addressed in longitudinal studies. In this context, it will be interesting to investigate the impact of (late-life) anxiety disorders on the findings, although (1) controlling for anti-anxiety medication might be challenging and impact the amygdala activity and (2) it is particularly challenging to recruit participants with high level of anxiety to a research study. Forth, and as mentioned above, we did not find strong differences in amygdala activation with emotional valence, which was against our hypotheses and previous findings. Most of studies investigated emotional processing in aging have used tasks that required manipulation of the emotional stimuli, which was not the case in our study. This discrepancy may explain some of these differences. Lastly, it is unclear why our significant results were largely focused on the left amygdala. There are a number of theoretical positions that hypothesize a lateralization effect on amygdala processing in both decision-making and emotional contexts (44–46). However, this data is highly inconsistent across neuroimaging studies and has at time been hypothesized to be a result of visual processing lateralization (47), sometimes related to sex (48), and often not found at all. It will be important to further investigate such lateralization differences in amygdala activation in future experiments with clear hypotheses in this regard.

## 5. Conclusion

This study aimed to investigate the pattern of variation in amygdala activation related to cognitive and affective traits during an affective Stroop task, in early and late adulthood. Our findings support the idea that the role of amygdala changes in its involvement in both affective and cognitive processing throughout adulthood. While it has been well accepted that the amygdala plays a major role in reactivity to negative stimuli and in reacting toward salient stimuli, it seems to be more sensitive during the early stages of life. The current study reinforces the importance of investigating the entire lifespan and particularly late adulthood, when investigating the link between behavior and brain features in relation to affective processing. Notably, the present findings suggest that the neurobiological correlates underlying anxiety may differ between early and late adulthood and therefore provide promising clues for future investigations of the neural mechanisms underlying late-life anxiety disorders.

## Data availability statement

The raw data supporting the conclusions of this article will be made available by the authors, without undue reservation.

## Ethics statement

The studies involving human participants were reviewed and approved by Institutional Review Board for Research with Human Subjects at Boys Town National Research Hospital. The participants provided their written informed consent to participate in this study.

## Author contributions

GD and SW contributed to conception and design of the study. NH and JK collected and organized the database. JK analyzed the neuroimaging data. JO and GD performed the statistical analyses. GD wrote the first draft of the manuscript. GD, SW, and JO wrote sections of the manuscript. All authors contributed to manuscript revision, read, and approved the submitted version.
