# Supplementary material for: Changing role of the amygdala in affective and cognitive traits between early and late adulthood
Source: Front Psychiatry. 2023 Feb 7;14:1033543. doi: 10.3389/fpsyt.2023.1033543 (PMC9941165; doi:10.3389/fpsyt.2023.1033543)
Supplement: Supplementary file 1 [file Data_Sheet_1.DOCX]

**Supplementary Material**


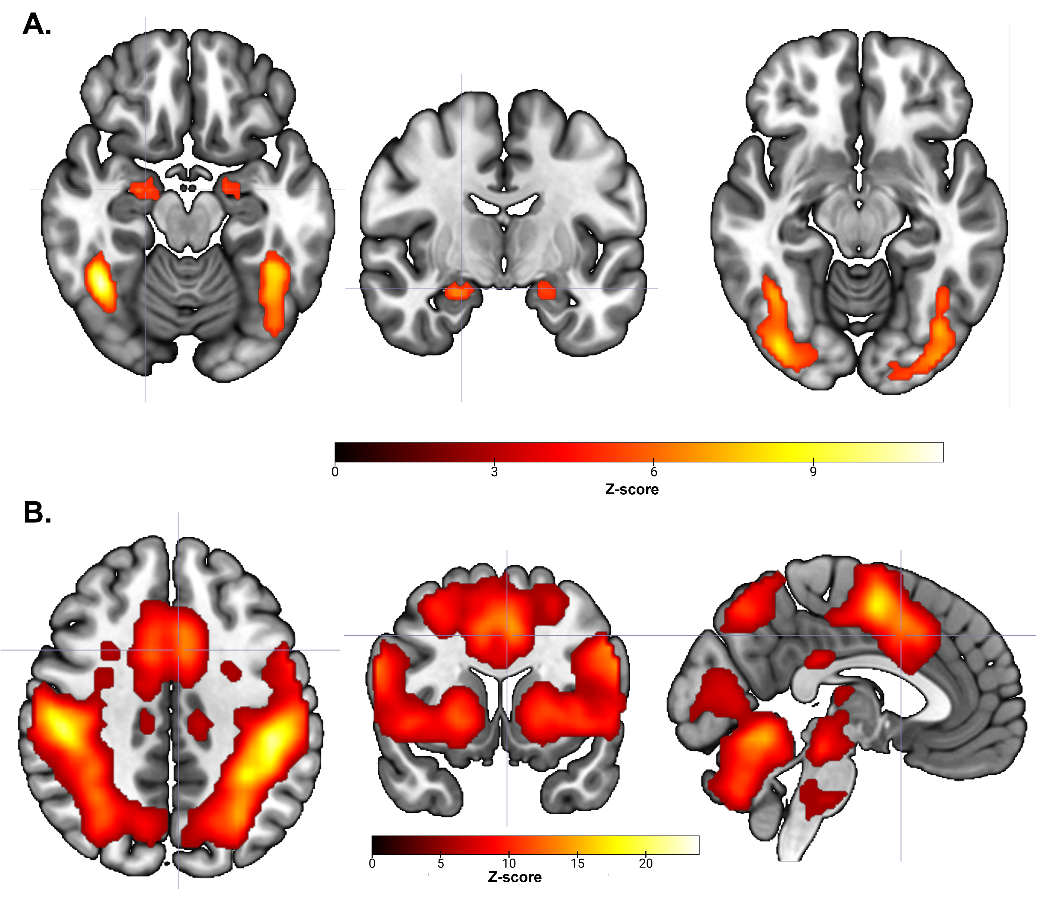


**Supplementary Figure S1:** Activation maps during the Affective Stroop Task, across all participants. (A) positive+negative view > neutral view; (B) congruent + incongruent trials > view. Statistical maps are displayed at p<0.05 (FWE, whole brain level), cluster size>10 voxels.


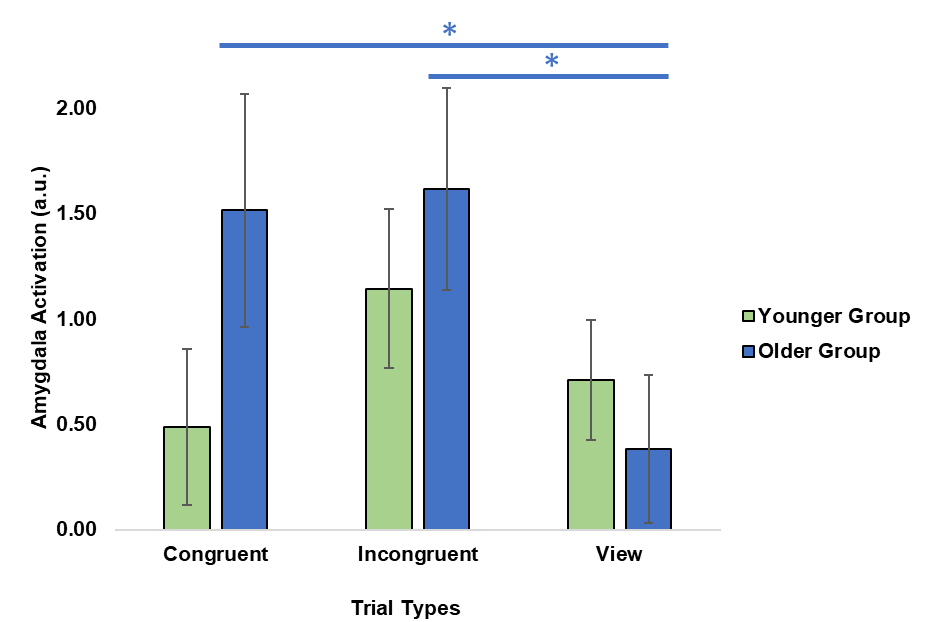


**Supplementary Figure S2:** Trial type by age group interactions for negative stimuli only. *: p<0.05 uncorrected.

**Supplementary Table S1:** Differences in activation during the congruent + incongruent trials > view trials contrast between the older and younger age groups.

| **Region** | **cluster size (voxel number)** | **p(FWE-corr, cluster level)** | **Z-scores** | **x** | **y** | **z** |
| --- | --- | --- | --- | --- | --- | --- |
| **Older > Younger Group** |  |  |  |  |  |  |
| Right postcentral gyrus | 1276 | 5.55E-15 | 5.59 | 42 | -18 | 42 |
| Right supramarginal gyrus |  |  | 5.55 | 51 | -27 | 33 |
| Right supramarginal gyrus |  |  | 4.76 | 63 | -18 | 24 |
| Left postcentral gyrus | 784 | 8.8E-11 | 5.45 | -42 | -18 | 48 |
| Left precentral gyrus |  |  | 5.08 | -33 | -12 | 51 |
| Left superior parietal cortex |  |  | 4.93 | -21 | -45 | 54 |
| Right cerebellum | 102 | 0.012028 | 4.68 | 9 | -60 | -12 |
| Right cerebellum |  |  | 3.84 | 30 | -54 | -30 |
| Right cerebellum |  |  | 3.80 | 18 | -48 | -21 |
| Left cerebellum | 132 | 0.003587 | 4.42 | -24 | -54 | -24 |
| Left cerebellum |  |  | 3.92 | -21 | -33 | -24 |
| Left cerebellum |  |  | 3.71 | -30 | -48 | -30 |
